# Evaluation of the Chinese Medicinal Herb, *Graptopetalum paraguayense*, as a Therapeutic Treatment for Liver Damage in Rat Models

**DOI:** 10.1155/2012/256561

**Published:** 2012-07-02

**Authors:** Li-Jen Su, Chih-Hsueh Yang, Shiu-Feng Huang, Ya-Ling Yuo, Hui-Chu Hsieh, Tzu-Ling Tseng, Chang-Han Chen, Shih-Lan Hsu, Chi-Ying F. Huang

**Affiliations:** ^1^Institute of Systems Biology and Bioinformatics, National Central University, Jhongli City 32001, Taiwan; ^2^Graduate Institute of Microbiology and Public Health, National Chung Hsing University, Taichung 40227, Taiwan; ^3^Division of Molecular and Genomic Medicine, National Health Research Institutes, Miaoli County, Zhunan 35053, Taiwan; ^4^Biomarker Technology Development Division, Biomedical Technology and Device Research Labs, Industrial Technology Research Institute, Hsinchu 31040, Taiwan; ^5^Center for Translational Research in Biomedical Sciences, Kaohsiung Chang Gung Memorial Hospital, Kaohsiung 83301, Taiwan; ^6^Department of Education and Research, Taichung Veterans General Hospital, Taichung 40705, Taiwan; ^7^Institute of Biopharmaceutical Sciences, National Yang-Ming University, Taipei 11221, Taiwan; ^8^National Yang-Ming University-VGH Genome Research Center, Taipei 11221, Taiwan

## Abstract

The incidence of cirrhosis is rising due to the widespread occurrence of chronic hepatitis, as well as the evident lack of an established therapy for hepatic fibrosis. In the search for hepatoprotective therapeutic agents, *Graptopetalum paraguayense* (GP) showed greater cytotoxicity toward hepatic stellate cells than other tested herbal medicines. Histopathological and biochemical analyses suggest that GP treatment significantly prevented DMN-induced hepatic inflammation and fibrosis in rats. Microarray profiling indicated that expression of most of metabolism- and cell growth and/or maintenance-related genes recovered to near normal levels following GP treatment as classified by gene ontology and LSM analysis, was observed. ANOVA showed that expression of 64% of 256 liver damage-related genes recovered significantly after GP treatment. By examining rat liver samples with Q-RT-PCR, five liver damage-related genes were identified. Among them, *Egr1* and *Nrg1* may serve as necroinflammatory markers, and *Btg2* may serve as a fibrosis marker. *Oldr1* and *Hmgcs1* were up- and down-regulated markers, respectively. A publicly accessible website has been established to provide access to these data Identification of 44 necroinflammation-related and 62 fibrosis-related genes provides useful insight into the molecular mechanisms underlying liver damage and provides potential targets for the rational development of therapeutic drugs such as GP.

## 1. Introduction

Hepatic fibrosis is a wound-healing process that follows chronic liver injury and is characterized by the activation of hepatic stellate cells (HSCs) and excess production of extracellular matrix (ECM) components. HSC activation involves transdifferentiation from a quiescent state into myofibroblast-like cells and includes the appearance of alpha smooth muscle actin (*α*-SMA) and loss of cellular vitamin A storage [1]. Activated HSCs are distinguished by accelerated proliferation and enhanced production of ECM components. Major cascades of the injury process have been reported, including interactions between damaged hepatocytes and Kupffer cell activation, HSC proliferation and activation and the excess production of ECM components during hepatic fibrosis [2]. However, the types of genes involved and their reactions to liver injury and healing are unclear. Moreover, cirrhosis caused by these risk factors often progresses insidiously. Patients with end-stage liver cirrhosis usually die without liver transplantation, which has a 75% five-year survival rate [3].

The identification of both effective drug treatments and molecular markers for liver fibrosis diagnosis is an important task. Numerous agents, including Silymarin and penicillamine, have been employed to treat hepatic fibrosis [4, 5]. Silymarin, a mixture of flavonoids extracted from the seeds of *Silybum marianum* containing three structural isomers (silybin, silydianin, and silychristin), has exhibited hepatoprotective effects both *in vivo* and *in vitro* [4]. Silymarin suppresses the expression of both profibrogenic procollagen alpha (I) and Timp1, most likely via downregulation of *Tgfb1*, in rat models [6]. It is also used to protect liver cell membranes against hepatotoxic agents and has been shown to improve liver function in both experimental animals and humans [7]. Corticosteroids and methotrexate may play roles in primary sclerosing cholangitis (PSC) treatment. Penicillamine is an effective treatment for Wilson's disease, which is characterized by excessive copper accumulation in the liver and other organs. However, due to Penicillamine's serious side effects, there is not yet a generally definitive treatment [8–11]. Herbal medicines have been used in China for thousands of years. Traditional Chinese herbal medicines may contain therapeutic agents for treating hepatic fibrosis [12]. Moreover, there is a growing trend in Western countries to use Chinese medicine to treat a wide range of diseases, such as inflammatory diseases and chronic liver diseases (including hepatitis and fibrosis). For example, Radix Polygoni Multiflori, the root tubers of *Polygonum multiflorum* Thunb, is a traditional Chinese treatment for liver disease and has been shown to impede the hepatic deposit of collagen and significantly improve survival rates in mice with DMN-induced liver cirrhosis [13]. Sho-saiko-to (TJ-9), a potent antifibrosis drug that inhibits lipid peroxidation in hepatocytes and HSCs, is an effective treatment for liver inflammation and fibrosis [14, 15]. Inchin-ko-to (TJ-135) is a possible treatment for liver fibrosis and portal hypertension that acts through suppression of activated HSC function by regulating PDGF-dependent events in HSCs and attenuating the development of liver fibrosis [16, 17]. *Graptopetalum paraguayense* (GP), a traditional Chinese medicine, has been identified as a possible hepatoprotective therapeutic agent. GP, which has been used as a health food in Taiwan, exhibits potentially beneficial effects on hypertension, diabetes, hyperuricemia, inflammation, and chronic liver diseases. However, the lack of information regarding these compounds' molecular mechanisms diminishes their clinical utilities.

This study aims to characterize the therapeutic effects of GP on liver fibrosis using microarray profiling. Silymarin was used as a positive drug control. Dimethylnitrosamine (DMN), a potent nongenotoxic hepatotoxin, has been demonstrated to induce liver damage rapidly and is empirically proven to be useful in the study of early human fibrosis formation [18]. We employed DMN to induce liver fibrosis in rats and performed a six-week time course Affymetrix microarray study [19]. A quantitative depiction of transcriptional regulation over the course of liver fibrosis was achieved using statistical analysis of histopathological grading of the rats. The histopathological, clinical biochemical, and microarray data are freely available at http://ehco.iis.sinica.edu.tw:8080/LFZ/.

## 2. Materials and Methods

### 2.1. Preparation of GP

GP was purchased from a herb farm in Taiwan. GP leaves were washed with distilled water and air dried overnight, then freeze-dried at −50°C by a frozen dryer, and ground into powder (100 mesh). Lyophilized GP powder was stored in a sealed container at 4°C until use. To prepare GP extracts, GP powder was first dissolved in water, and ethanol was gradually added to a final concentration of 80%. After centrifugation at 1400 ×g for 20 min, the resulting precipitates were discarded; the supernatant was filtered through a 0.22 *μ*m filter and evaporated to dryness on a rotary evaporator. The recovery of GP extracts after ethanol treatment was 65%. The dried extracts were dissolved in water at a concentration of 50 mg/mL as a stock solution. The stock solution was then diluted in culture medium to the appropriate working solution.

### 2.2. Isolation of HSCs and Cell Viability Determination

HSCs were isolated from the livers of male SD rats (500 to 600 g). Briefly, the livers were perfused *in situ* through the portal vein with a 16-guage cannula, first with Ca^2+^/Mg^2+^-free HBSS solution at 37°C for 10 min at a flow rate of 10 mL/min, followed by 0.1% pronase E (Merck, Darmstadt, Germany) in HBSS solution for 10 min and 0.3% collagenase (Wako, Osaka, Japan) in HBSS solution for 30 min. The digested liver was excised, minced with scissors, and incubated in HBSS solution containing 0.05% pronase E and 20 *μ*g/mL DNase for 30 min. The resulting suspension was filtered through a nylon mesh (150 mm in diameter). An HSC-enriched fraction was obtained by centrifugation of the filtrate in an 8.2% Nycodenz (Nycomed, Oslo, Norway) solution at 1400 ×g and 4°C for 20 min. The cells in the upper layer were washed by centrifugation at 450 ×g at 4°C for 10 min and suspended in DMEM supplemented with 10% fetal bovine serum, 100 U/mL penicillin, 100 *μ*g/mL streptomycin, and 1% L-glutamine. The purity of the isolated HSCs was assessed both by direct cell counting under phase-contrast microscopy using intrinsic vitamin A autofluorescence and by immunohistochemistry using a monoclonal antibody against desmin (DAKO; diluted 1 : 40). Cell viability was examined using trypan blue dye exclusion. Both cell purity and viability were in excess of 90%. HSCs were plated at a density of 5 × 10^5^ cells per well in 1 mL of culture medium on uncoated plastic culture dishes. The culture medium was changed 2 days after plating. GP extracts were first prepared as PBS stock solutions (0.5 g/mL) and then diluted in culture medium to the appropriate working solutions. Cells were maintained at 37°C in a 5% CO_2_ incubator for indicated time periods.

### 2.3. Animal Treatments

Liver fibrosis in the rat models was induced using DMN as previously described [19]. DMN treatments took place over three weeks (Figure 2(a)). GP extracts (1.4 g/kg) or Silymarin (0.4 g/kg) was orally administered daily to the rats beginning on day 7 of DMN treatment. All rats received distilled water and libitum. Food intake and body weights were recorded weekly, and rats were observed daily for clinical signs of ill health. Rats were euthanized with CO_2_. All animals that were euthanized or found dead were autopsied, and their tissues were fixed in 10% neutral buffered formalin. Rats were separated into groups of 4 to 7, and each group was treated with either DMN or an equal volume of normal saline without DMN as a control. All rats were subjected to biochemical and histopathological analyses. Two rats from each group were subjected to microarray analysis every week. Rats were weighed and euthanized on days 11, 18, 25, 32, 39, and 46, designated as weeks 1 through 6, respectively (Figure 2(a)).

### 2.4. Histopathological Examination

Liver specimens were fixed with phosphate-buffered formaldehyde, embedded in paraffin, and stained with hematoxylin-eosin. Differential staining of collagenous and noncollagenous proteins was performed with 0.1% Sirius red and 0.1% fast green as a counterstain in saturated picric acid, resulting in red-stained collagens. The scoring system, based on the histology activity index (HAI) [20, 21], included necroinflammatory, fibrosis, and fatty change scores as previously described [19]. Three images of each histology sample section (at 100× magnification) from each rat were selected randomly, scored, and deposited on the publicly accessible website (http://ehco.iis.sinica.edu.tw:8080/LFZ/). 

### 2.5. Serum Biochemical Data

Blood samples collected from the animals at autopsy were used to measure serum concentrations or activities of albumin, aspartate aminotransaminase (AST), alanine aminotransferase (ALT), total bilirubin, acid phosphatase (ACP), *α*-fetoprotein (AFP), blood urea nitrogen (BUN), lactate dehydrogenase (LDH), globulin, prothrombin time (PT), and blood platelets (PLT) using a Hitachi 747 and an ACL 3000 clinical chemistry analyzer system (MYCO, Renton, Washington) at Taichung Veterans General Hospital, Taiwan.

### 2.6. RNA Extraction, Reverse Transcription, and Quantitative Real-Time-Reverse Transcriptase-Polymerase Chain Reaction (Q-RT-PCR)

The same total RNA samples were used for both microarray and Q-RT-PCR analyses, which were performed as previously described [19]. Applied Biosystems Assays-on-Demand was used to identify transforming growth factor beta-1 (*Tgfb1*), tissue inhibitor of metalloproteinase 1 (*Timp1*), tissue inhibitor of metalloproteinase 2 (*Timp2*), peroxisome proliferator-activated receptor gamma (*Pparg*), B-cell translocation gene 2 (*Btg2*), early growth response 1 (*Egr1*), oxidized low-density lipoprotein (lectin-like) receptor 1 (*Olr1*), neuregulin 1 (*Nrg1*), 3-hydroxy-3-methylglutaryl-Coenzyme A synthase 1 (*Hmgcs1*), and 18s ribosomal RNA (as an internal control).

### 2.7. Microarray Analysis, Data Analysis, and Clustering Algorithm

Protocols and reagents for hybridization, washing, and staining followed Affymetrix instructions (http://www.affymetrix.com/support/technical/manuals.affx). The images were transformed into text files containing intensity information using Affymetrix GeneChip Operating Software. Microarray datasets were then analyzed using GeneSpring 7.3.1 software (Silicon Genetics, Redwood City, CA).

### 2.8. Statistical Analysis

All statistical analyses were performed using SAS/STAT 8e (SAS Institute, Cary, NC). Biochemical data are expressed as mean ± standard deviation (mean ± S.D.). Two-way analysis of variance (ANOVA) was used to build an explicit model of the sources of variances in the measurements. The least squares means (LSM) method was used to identify significant differences among groups across treatment and time course factors. Differences between control groups and treated groups were evaluated on the basis of mean biochemical data from the first and the second weeks, the third and the fourth weeks, and the fifth and the sixth weeks. Similarities between Q-RT-PCR and microarray data of *Timp1*, *Tgfb1*, and *Pparg* are presented using Pearson's correlation coefficients. Necroinflammatory and fibrosis-associated genes were identified by statistical analysis. LSM, separately estimated for variations in each three-subgroup variations by necroinflammatory score, were used for necroinflammatory-related analysis. Nonparametric test methods, estimated for only two-subgroup variations by fibrosis score, were used in fibrosis-related analyses. Values of *P* < 0.05 were considered statistically significant.

## 3. Results

### 3.1. GP Has Greater HSC Cytotoxicity Than Other Tested Herbal Medicines

**Figure 1:**
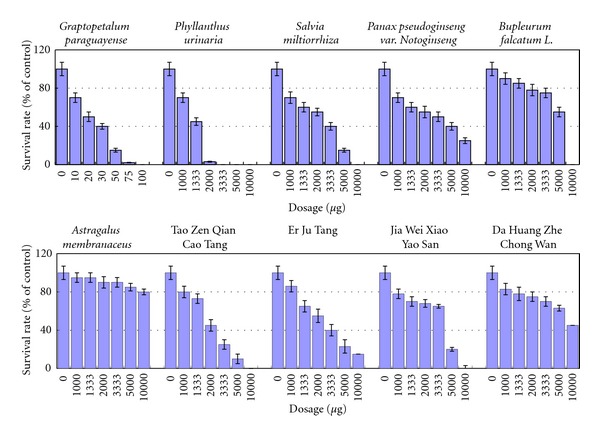
Screening of traditional Chinese medicines for therapeutic agents targeting liver fibrosis using HSC as a model. Characterizations of medicine-induced HSC cell death are shown. Ten traditional Chinese medicines, including six herb species and four formulas, were used: *Graptopetalum paraguayense*, *Phyllanthus urinaria*, *Salvia miltiorrhiza*, *Panax pseudoginseng var. Notoginseng*,* Bupleurum falcatum L.*, *Astragalus membranaceus*, Tao Zen Qian Cao Tang, Er Ju Tang, Jia Wei Xiao Yao San and Da Huang Zhe Chong Wan.

Herbal medicines have been used in Chinese population for thousands of years. It is generally believed that traditional Chinese herbal medicines may contain therapeutic agents for treating hepatic fibrosis [12]. Moreover, there has been a growing trend in Western countries to use Chinese herbal medicines to treat a wide range of diseases, such as inflammatory diseases and chronic liver diseases (including hepatitis and fibrosis). We tested HSC drug sensitivity using 10 traditional Chinese medicines (Figure 1), including six individual herb species (*Graptopetalum paraguayense, Phyllanthus urinaria, Salvia miltiorrhiza, Bupleurum falcatum L., Panax pseudoginseng *var*. Notoginseng*, and* Astragalus membranaceus*) and four formulas, (Tao Zen Qian Cao Tang, Er Ju Tang, Jia Wei Xiao Yao San, and Da Huang Zhe Chong Wan). Among the tested substances, HSCs were the most sensitive to GP. Next, we measured the expression of the activated HSC markers *α*-SMA and collagen 1; these were also downregulated after GP treatment in the culture system (see below, Figure 3(b)). We therefore focus on characterization of GP's therapeutic potential.

### 3.2. GP Serves as a Better Therapeutic Agent Than Silymarin for DMN-Induced Necroinflammation and Hepatic Fibrosis as Indicated by Histopathological and Biochemical Data

**Figure 2:**
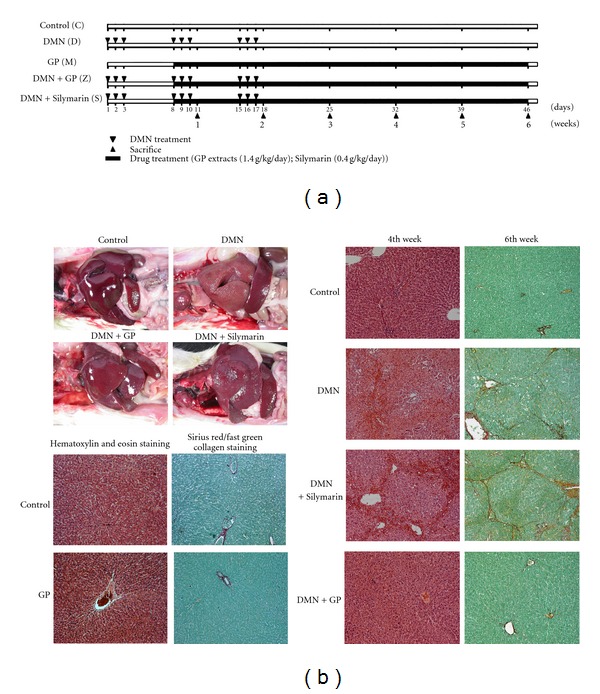
Histopathological analysis of the therapeutic effects of *Graptopetalum paraguayense* (GP) and Silymarin on liver damage in rat models. (a) Schematic illustration of DMN-induced fibrosis in rats. Each rat was injected with either DMN or saline, as a control, three times per week for three consecutive weeks (triangles). Subsequently, either Silymarin or GP was dissolved in water and given orally from days 8 to 46 (black rectangles). Rats were weighed and euthanized each week (starting on day 11, which begins what are referred to as the first through sixth weeks). Blood samples were collected for biochemical analysis (summarized in Table 1), and livers were excised and weighed, followed by either fixing in formaldehyde for histopathology or isolation of RNA for microarray analysis. (b) Anatomical evaluation of damaged livers (left upper panel). Based on exterior views, the GP had greater therapeutic effect than Silymarin. Representative phenotypes of DMN-induced rat liver fibrosis and therapeutic effects of GP and Silymarin were characterized by histopathological scoring. The fixed liver samples were then processed for paraffin embedding and prepared for hematoxylin and eosin staining (to score necroinflammation in the fourth week) and for Sirius red/fast green collagen staining (to score for fibrosis in the sixth week) (right panel). Drug toxicity effects were also evaluated by scoring. No notable liver damage patterns were observed after the six-week course of treatment (left lower panel). The original magnification was 100×.

We compared the therapeutic effects of GP and Silymarin on rats with DMN-induced liver fibrosis. A schematic illustration of the course of administration of DMN, GP, and Silymarin is shown in Figure 2(a). GP treatment resulted in increased survival rates in pilot studies (data not shown). The livers of GP-treated rats externally appeared much healthier than those treated with Silymarin, during liver damage (Figure 2(b)). To further delineate the biological and histopathological characteristics of GP treatment, experimental rats were weighed and euthanized and their livers excised and weighed. A scoring system, as previously described [19], was used to characterize phenotypic changes due to DMN-induced liver damage. Treatment with DMN caused significant decreases in both rat body and liver weights that were alleviated by GP treatment (data not shown). We next investigated GP's effect on necrosis and inflammatory responses in the liver following DMN exposure. Massive necrosis in the pericentral and midzonal area, with infiltration of inflammatory cells, was observed 3 weeks following DMN treatment. Following GP treatment, histopathological examinations found a 31% reduction in necroinflammatory response in the third and forth weeks and a 30% reduction of bridging fibrosis in the fifth and sixth weeks. These results indicated greater therapeutic value than those of Silymarin (19% reduction in necroinflammatory response and no effect on bridging fibrosis) (Table 1) and that GP treatment may significantly reduce DMN-induced hepatic fibrosis and necroinflammatory responses.

The sera of the control and DMN-treated rats both treated and untreated GP were subjected to various biochemical examinations. The rats were further divided into three subgroups (first to second weeks, third to fourth weeks, and fifth to sixth weeks) for statistical analysis. Biochemical analyses of the GP-treated subgroups indicated significant recovery compared to the DMN-treated groups as illustrated in Tables 2 and 3. Two-way ANOVA at a 5% significance level was performed to distinguish the variations between the treatments (e.g., DMN + GP versus DMN alone or differences due to the time course) and to estimate the variance of each individual variable in the ANOVA model. In the GP-treated groups, 9 of 14 serum markers, including ALT, AST, bilirubin, AKP, AFP, BUN, PT, and PLT, showed significant differences. These differences were not due to changes over the time course (1–6 weeks). These data indicate that DMN-induced increases in serum GTP, AST, and bilirubin levels were negated through GP treatment. Treatment with DMN resulted in marked reductions of serum albumin levels, but recovery at these levels was observed in the DMN + GP-treated groups. Although there were also significant effects on LDH, globulin, and ACP after GP treatment, two-way ANOVA indicated that changes in these three serum markers could be due to the time factor during 6-week experiments. Taken together, these pathological and biochemical data suggest that GP may have therapeutic effects on liver damage in rat models.

### 3.3. GP Suppresses *α*-SMA Expression in DMN-Treated Rats

**Figure 3:**
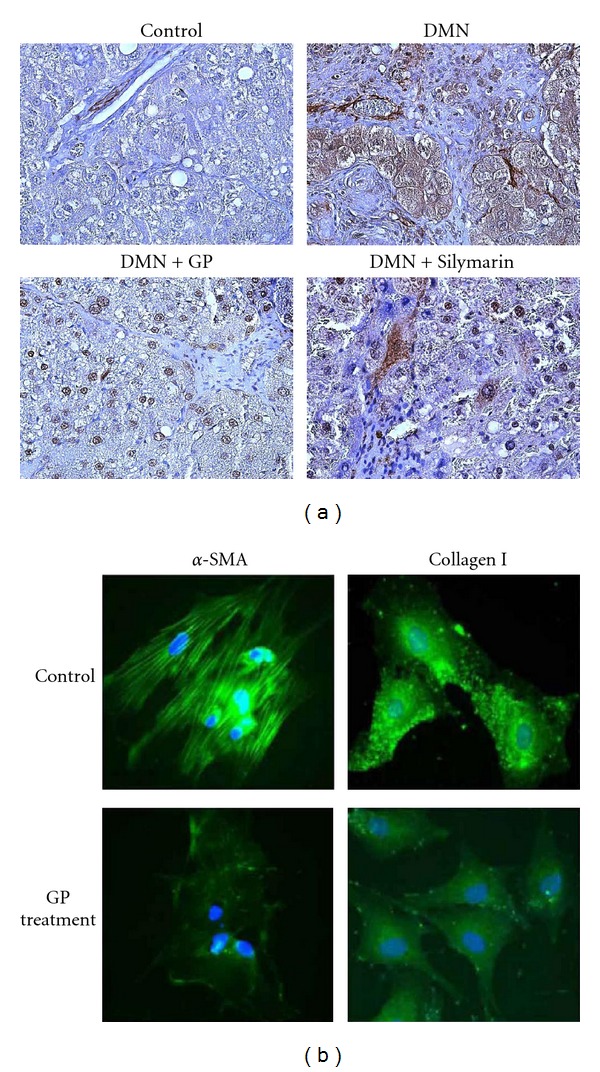
GP-derived reduction of DMN-induced *α*-SMA expression in livers and HSCs. Liver samples were excised 6 weeks after the first DMN injection. (a) The expression and localization of *α*-SMA in the liver were detected by immunohistochemical staining using a monoclonal antibody for *α*-SMA. Representative photomicrographs from controls, DMN treatment alone, DMN − Silymarin treatment, and DMN − GP treatment are shown. The original magnification was 100×. (b) Immunocytochemical staining of *α*-SMA and collagen I expression in cultured HSC. Cells were treated with or without 0.5 mg/mL GP extract for indicated time points and stained with FITC-conjugated *α*-SMA and collagen I antibodies. Representative photomicrographs from controls, DMN treatment alone, DMN − Silymarin treatment, and DMN − GP treatment are shown. The original magnification was 400×.

Next, the expression of *α*-SMA, an activated HSC marker, in the liver was measured by IHC staining 6 weeks after initial DMN administration. As expected, intense specific staining for *α*-SMA was observed in the pericentral area of the DMN-treated livers (Figure 3(a)). This expression was suppressed dramatically by oral administration of GP. Staining did not detect *α*-SMA in livers from vehicle control group rats or rats given GP or Silymarin alone, as expected (Data not shown). These findings indicate that GP prevented fibrogenic responses in the liver following DMN administration. In addition, TUNEL staining of histologic sections indicated an increase in the number of TUNEL-positive cells in GP-treated rat liver in regions containing *α*-SMA. Dual staining for TUNEL and *α*-SMA implied colocalization. These data suggest that the observed reduction in the number of activated HSCs in fibrotic liver in response to GP was mediated via apoptosis (data not shown).

### 3.4. Inhibitory Effect of GP on the Activation of Cultured Rat HSCs

Immunocytochemical analysis confirmed that GP suppressed *α*-SMA expression and stress fiber formation. HSCs were the major cellular source of extracellular matrix in hepatic fibrosis and were transformed into myofibroblast-like cells specifically expressing *α*-SMA. To examine the direct effect of GP on HSC activation, primary cultured HSCs were incubated with various doses of GP for 5 days (from days 5 to 10), and the expression of *α*-SMA was measured by indirect immunofluorescence analysis (Figure 3(b), left panel). At day 10 of culture, control HSC cultures demonstrated increased expression of *α*-SMA, which appeared to form stress fiber. However, continuous administration of GP for 5 days drastically inhibited *α*-SMA expression compared to these controls. Next, the effect of GP on expression of collagen I in activated HSCs *in vitro* was evaluated by immunocytochemical staining. At day 10, marked expression of collagen I in the cytoplasm of the control HSCs was observed (Figure 3(b), right panel). This expression was inhibited by the addition of GP extract. These observations confirmed that GP inhibited transactivation and production of *α*-SMA and collagen I in primary cultured HSCs *in vitro*.

### 3.5. GP Reverses Gene Expression Patterns of Liver-Damage-Related Markers

**Figure 4:**
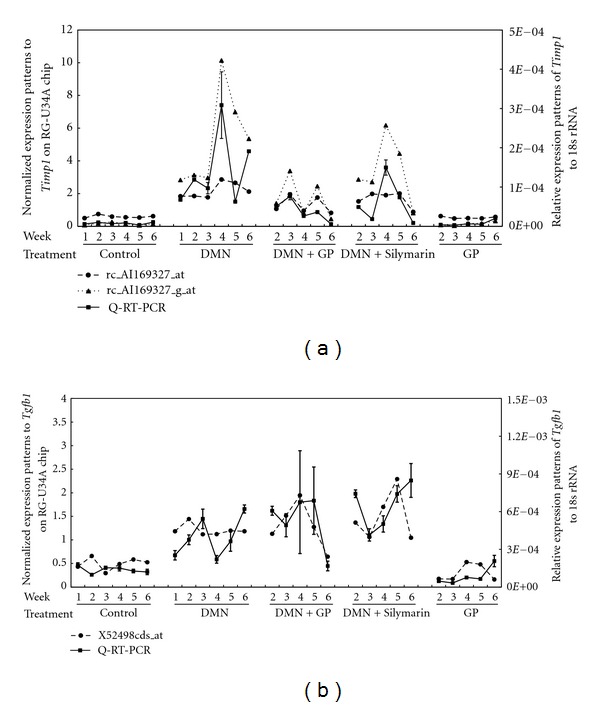
The therapeutic effects of GP were validated using marker expression patterns. A comparison of gene expressions measured by Q-RT-PCR and microarray is shown (a and b). TaqMan^®^ assays were conducted in triplicate for each sample, and the mean value was used to calculate expression levels (square markers). To standardize the quantification of *Timp1* (a) and *Tgfb1* (b), 18s rRNA from each sample was quantified at the same time as the target gene and is shown on the right-hand log scale. For the *Timp1* transcripts, rc_AI169327_at and rc_AI169327_g_at (circles and triangles, resp., in a) and *Tgfb1* transcripts, X52498cds_at (triangles in b), expression levels from microarray data are relative to the average of all gene expression levels and are shown on the left-hand scale.

Previous biochemical studies have identified several well-known fibrosis and cirrhosis markers, both invasive and noninvasive [3, 22, 23]. For example, tissue inhibitors of metalloproteinases (TIMPs), secreted by activated HSCs, can prevent matrix degradation by inhibiting the enzymatic activities of matrix degrading metalloproteinases (MMPs) [3] and transforming growth factor beta-1 (TGFB1). TGFB1 is also the strongest known inducer of fibrogenesis in the effecter cells of hepatic fibrosis and can stimulate the adipocyte transformation [24–27]. To study the molecular characteristics of liver fibrosis in DMN-treated rats and the therapeutic efficacy of GP, we examined the gene expression profiles of rat liver samples using Affymetrix rat oligochips (RG-U34A) and Q-RT-PCR analysis. Consistent with previous observations, both microarray and Q-RT-PCR indicated higher levels of *Tgfb1* and *Timp1* mRNA expression in DMN-treated rat livers than in the controls (Figures 4(a) and 4(b)). Expression of these markers was significantly reduced when the DMN-damaged rats were treated with GP, but only *Timp1* expression was reduced when Silymarin was administered. Regulation of peroxisome proliferator-activated receptors (PPARs), a transcription factor family involved in the retinoic-acid- (RA-) mediated signal pathway of lipid metabolism [28], usually takes place when HSCs are activated in culture systems [29]. In this study, downregulated expression of *Pparg* was found in DMN-treated rat liver, consistent with the hypothesis that downregulation of *Pparg* may be connected to liver inflammation and fibrosis mechanisms (see Supplementary Figure  1 available online at doi: 10.1155/2012/256561). Microarray and Q-RT-PCR results indicated that *Pparg* expression recovered after GP treatment, suggesting potential for protecting or preventing liver damage.

### 3.6. Microarray Analysis

**Figure 5:**
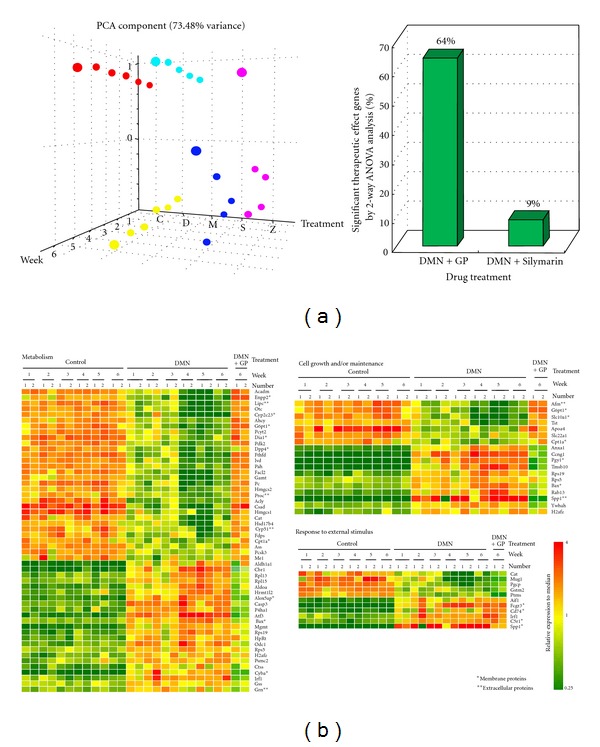
The 256 gene expression patterns from experimental samples. (a) Principal component analysis (PCA) of liver damage-related gene expression profiles from 5 different groups, including normal (C, marked in red), DMN treatment (D, yellow), GP treatment only (M, cyan), Silymarin-therapeutic (S, blue), and GP-therapeutic (Z, pink). PCA analyses were conducted on the expression of the 256 genes on the array (left side). The 3D distribution patterns indicate that there were excellent therapeutic effects and no notable liver damage patterns at molecular levels. Based on the ANOVA results (right), expression of 64% of the genes (165 genes out of 256) was significantly reversed in the GPtherapeutic treatment, whereas only 9% (23 genes) were reversed using Silymarin. (b) Hierarchical clustering identified three biological process categories: metabolism, cell growth and/or maintenance and response stimulus. Here, rows represent individual transcripts and columns represent each time course sample. The right two columns of each classification indicate the expression patterns of the GP therapeutic groups in the 6th week. Expression of metabolism and cell growth and/or maintenance genes in the GP therapeutic groups reverted to normal levels; response stimulus gene expression did not. The color in each cell reflects the expression level of the corresponding sample relative to its mean expression level, and the scale extends from fluorescence ratios of 0.25 to 4 relative to the mean level for all samples.

A set of 256 previously identified liver damage-related discriminator genes [19] were further analyzed using principal component analysis (PCA), a decomposition technique that produces a set of expression patterns known as principal components. Rats in the DMN-damaged group treated with GP fared much better than those treated with Silymarin, especially in the sixth week (Figure 5(a), left), in this analysis. Hierarchical clustering found similar results (Supplementary Figure  2).Additional ANOVA found that the expression of 64% of the 256 genes recovered significantly after GP treatment, but only 9% recovered after Silymarin treatment (Figure 5(a), right). Significantly, treatment with GP returned the expression of more than 60% of these discriminators to the levels of the controls. The 256 genes were further classified via gene ontology analysis (http://www.fatigo.org/) [30]. In each category, the largest group (approximately 50%) was found to be uncharacterized genes in the summary results (data not shown). Hierarchical clustering was further employed to organize each of the top three biological process categories into a dendrogram. Interestingly, with the exception of stress stimulus-related genes, most of the metabolism- and cell growth and/or maintenance-related genes recovered to near normal liver expression levels (Figure 5(b)). Identification of these discriminators allows them to serve as molecular classifiers, providing novel biological insights into the early development of liver damage, and aids in the development of new therapeutic drugs for liver disease.

Overall expression patterns for the 2,409 microarray transcripts (8,799 probe sets analyzed) were further analyzed for evidence of GP therapeutic action. GP treatment-related genes were clustered using two-way ANOVA at a 5% significance level to distinguish the various variations (e.g., GP treatment with DMN-damage versus only DMN-damage and differences due to the time course) and to estimate the variance of each individual variable in the ANOVA model. The resulting 813 transcripts gave rise to distinct molecular signatures of liver fibrosis progression that could be partly reversed GP treatment. Out of the 813 genes, 168 were also present in the list of 256 liver damage-related genes and reverted to normal levels significantly after treatment with GP or Silymarin (Supplementary Table  3). Interestingly, expression of 145 of the 168 genes was significantly reversed by GP treatment but not by Silymarin. In contrast, the expression of only 3 genes was significantly reversed by Silymarin but not by GP. Twenty genes were reversed by both GP and Silymarin, suggesting that they share same expression mechanism. Statistical analysis indicates that these 168 genes might serve as therapeutic target genes and GP could regulate the gene expression patterns better than Silymarin.

### 3.7. Necroinflammatory and Fibrosis-Related Gene Expression Profiling

**Figure 6:**
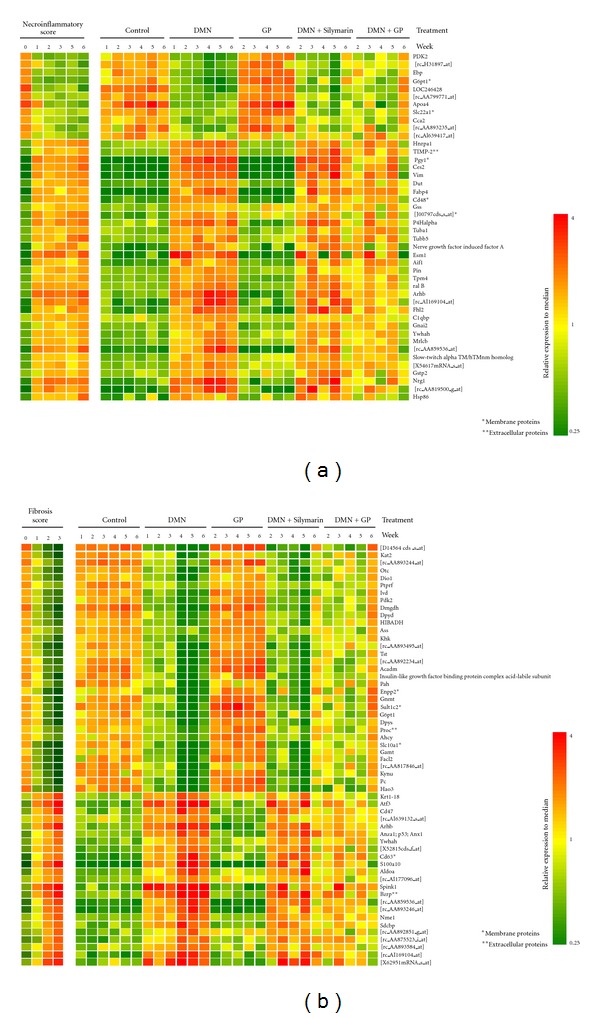
Hierarchical clustering illustrates necroinflammatory and fibrosis-related gene expression patterns. Rows represent individual transcripts and columns represent time course samples. Transcripts were ranked using hierarchical clustering of necroinflammatory (a) and fibrosis scores (b). Colors reflect the expression level of the each sample relative to its mean expression level.

It has been suggested that necroinflammation and fibrosis play important roles in liver cirrhosis progression in rat models [18–22, 31]. To clarify the factors responsible for this histopathological phenotype, all histopathological samples were scored for necroinflammation and fibrosis as previously reported [19]. The mRNA expression levels of 44 genes, assayed by microarray, were significantly correlated with unchanged to higher scores according to a statistical analysis that separately estimated variations in necroinflammatory score and the 62 differentially expressed genes in fibrosis score, as previously reported [19]. These discriminators are shown in the left panels of Figures 6(a) and 6(b) (necroinflammation and fibrosis, resp.) and plotted over the time course for each group (Figures 6(a) and 6(b), right panel). Hierarchical clustering analysis indicates that gene expression reservation patterns after GP treatment were much closer to those of normal liver than those after Silymarin.

### 3.8. Q-RT-PCR Validations for Novel Liver Damage Markers

**Figure 7:**
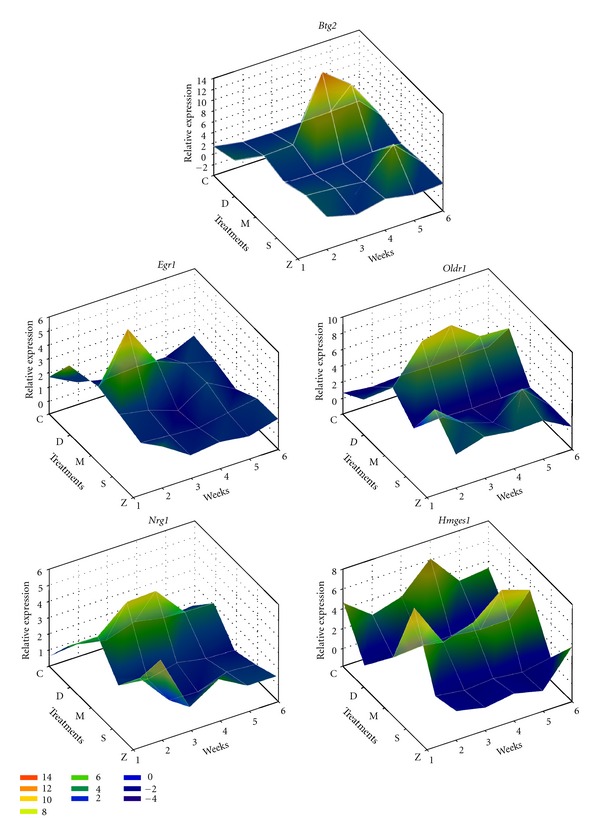
Q-RT-PCR validation of novel therapeutic markers. Five candidate markers, *Btg2*, *Egr1*, *Oldr1*, *Nrg1*, and *Hmgcs1*, were validated by Q-RT-PCR and are illustrated on 3D mesh plots. 18s rRNA from each sample was quantified at the same time as the target gene. *Btg2*, *Egr1*, *Oldr1*, *Nrg1* were upregulated with liver damage and recovered following both drug treatments. However, *Hmgcs1* was downregulated and recovered after GP administration only. The five different groups are shown as normal (C), DMN treatment (D), GP treatment only (M), Silymarin therapeutic (S), and GP-therapeutic (Z).

From Q-RT-PCR assays of rat liver samples, 5 novel liver damage-related genes, including *Btg2*, *Egr1*, *Oldr1*, *Nrg1*, and *Hmgcs1*, were subjected to further statistical analysis (Figure 7). An internal control, 18 s ribosomal RNA, was used to obtain relative expression patterns and for comparison. A high Pearson correlation was found between the microarray and Q-RT-PCR results. Based on the expression patterns, *Egr1* and *Nrg1* may act as necroinflammatory markers, and *Btg2* may act as a fibrosis-related marker. *Oldr1* and *Hmgcs1* were up- and downregulated markers for liver damage, respectively. These genes show potential as commercial diagnostic markers.

## 4. Discussion

The gene expression profiling described here provides a powerful and robust tool that is able to reveal molecular markers for liver damage at an early stage. Although several liver damage-related gene expression profiling analyses have been reported in animal models [32, 33], these gene discriminators have rarely been applied for therapeutic purposes. However, the identification of a drug treatment that improves outcomes for patients with liver fibrosis and molecular markers for liver fibrosis diagnosis are important therapeutic needs. Comparison of our dataset with earlier related studies reveals multiple overlapping gene identities that may potentially serve as markers for fibrosis, cirrhosis, and/or HCC diagnosis [19]. The expression patterns enabled us to identify 256 differentially expressed genes, including 44 necroinflammatory and 62 fibrosis-related genes. To our knowledge, this is the first paper to delineate the therapeutic effects of GP, ranging from histopathologic and biochemical analyses to a molecular portrait of liver fibrosis based on the courses of necroinflammation and fibrosis over time. Our findings suggest that GP has excellent anti-inflammatory, hepatoprotective, and antifibrotic activities and has potential as a novel antifibrotic medication.

CD63, a transmembrane protein, which was upregulated after DMN treatment, was identified as a fibrosis gene signature. Inhibition of CD63 inhibits collagen secretion and HSC migration [34]. In an alcoholic liver disease (ALD) study [35], Annexin A1 (*Anxa1*) was highly expressed after liver injury. A recent study has indicated that alcohol-initiated liver injury occurs via inflammation. ALD progression involves continuing liver injury, fibrosis and impaired liver regeneration. In the current study, the expression patterns of both *Cd63* and *Anxa1*, both of which were present in the list of potential therapeutic target genes, significantly recovered following GP, but not Silymarin, treatment. Extending the previous study [19], identification of these necroinflammatory and fibrosis-related genes provides useful insight into the molecular mechanisms underlying liver damage and provides potential targets for the rational development of therapeutic drugs such as GP.

Although limited by the complex cellular components, there were still dramatic damage-related patterns present. For example, early growth response 1 (Egr1) was an immediate-early gene transcription factor and was identified as an important contributor to increased LPS-stimulated TNF-alpha secretion by Kupffer cells after chronic ethanol exposure [36]. Egr1 also controls the expression of a number of inflammatory mediators contributing to liver fibrosis. Based on our observations, Egr1 expression in DMN-damaged liver was higher than that in normal liver, but the expression levels recovered to near normal after GP treatment (Figure 7). These results indicate that Egr1 may be an effective therapeutic target in the treatment of liver damage.

Models for fibrosis were first developed in rodents. Carbon tetrachloride (CCl_4_) [37] and DMN are the most commonly used hepatotoxic agents, and they ensure bridge fibrosis first developed in pericentral areas and secondarily between central and portal areas eventually leading to cirrhosis. Another secondary biliary fibrosis model is common bile duct ligation (BDL) [38]. However, it is obvious that they are not human and there are large species differences, for example, pharmacological, metabolic, or tissue responses. The whole organ is more intact than *in vitro* observation system, and the animal models still are better way and allow for comprehensive study of questions that cannot be addressed in human studies, especially in drug development. In addition, the preliminary results indicated that GP also diminished CCl_4_- and BDL-induced fibrosis and effectively alleviated fibrogenic progression by image morphometric analysis (data not shown).

Currently available human hepatic fibrosis treatments remain unsatisfactory due to a lack of antifibrosis agent selectivity against fibrogenesis. GP is a potent candidate for development as an antifibrosis drug. Despite GP's attractive antifibrotic properties, its safety has not been fully evaluated. GP toxicity in animal models requires further study to move the substance toward human clinical trials. Acute toxicity effects demonstrate the relationship between dosage and the proportion of individuals responding with a quantifiable effect such as death. The half-lethal dose (LD_50_), the slope of the lethality curve, and prominent clinical effects are all required to characterize the potency of the drug. GP's acute toxicity was analyzed to determine these characteristics (data not shown). In future studies, we will evaluate the therapeutic effects of GP on liver-fibrosis in more detail and in comparison to clinical applications of GP. The mechanism and active components of GP require further study.

## Supplementary Material

Supplementary Figure 1: Down-regulated expression of peroxisome proliferator-activated receptor, gamma (*Pparg*) was found in DMN-treated rat liver, consistent with the hypothesis that down regulation of *Pparg* may be connected to liver inflammation and fibrosis mechanisms. Microarray and Q-RT-PCR results indicated that *Pparg* expression recovered after GP treatment, suggesting potential for protecting or preventing liver damage.
Supplementary Figure 2: Rats in the DMN-damaged group treated with GP fared much better than those treated with Silymarin, especially in the sixth week by hierarchical clustering analysis.
Supplementary Table: Statistical analysis indicates that these 168 genes might serve as therapeutic target genes and GP could regulate the gene expression patterns better than Silymarin.

## Figures and Tables

**Table 1:** Summary of rat model histopathological scores.

Factor (scores)		DMN			DMN + GP			DMN + Silymarin			GP	Control
		1-2 wk	3-4 wk	5-6 wk	2 wk	3-4 wk	5-6 wk	2 wk	3-4 wk	5-6 wk	2–6 wk	1–6 wk
		*n* (%)	*n* (%)	*n* (%)	*n* (%)	*n* (%)	*n* (%)	*n*(%)	*n* (%)	*n* (%)	*n* (%)	*n* (%)
Necroinflammatory	A0	0 (0)	1 (9)	4 (44)	3 (50)	4 (45)	9 (64)	0 (0)	2 (22)	2 (20)	10 (100)	100 (24)
	A (1–3)	5 (62.5)	3 (27)	4 (44)	3 (50)	2 (22)	1 (7)	3 (75)	3 (33)	2 (20)	0 (0)	0 (0)
	A (4–6)	3 (36.5)	7 (64)	1 (12)	0 (0)	3 (33)	4 (29)	1 (25)	4 (45)	6 (60)	0 (0)	0 (0)

Fibrosis	F (0-1)	6 (75)	1 (9)	2 (22)	6 (100)	8 (67)	7 (50)	3 (75)	3 (33)	0 (0)	10 (100)	100 (24)
	F (2-3)	2 (25)	10 (91)	7 (78)	0 (0)	4 (33)	7 (50)	1 (25)	6 (67)	10 (100)	0 (0)	0 (0)

Fatty change	−	7 (87.5)	11 (100)	9 (100)	6 (100)	12 (100)	14 (100)	4 (100)	8 (89)	10 (100)	10 (100)	100 (24)
	+	1 (12.5)	0 (0)	0 (0)	0 (0)	0 (0)	0 (0)	0 (0)	1 (11)	0 (0)	0 (0)	0 (0)

Results are ranked by time course. Necroinflammatory change was scored as A (0), no change; A (1–3), mild; A (4–6), moderate necroinflammation. Fibrosis was graded as F (0-1) for observations ranging from normal tissue to fibrous expansion of portal tracts and F (2-3) for bridge fibrosis to frequent bridging fibrosis with nodule formation. The fatty change was classified by its presence or absence (+/−). The number of rats was counted and used to calculate the percentage of each histopathological level at each time point.

*n*: number of rats.

**Table 2:** Therapeutic effects of *Graptopetalum paraguayense* (GP) on DMN-induced liver fibrosis in rats via biochemical analysis (1).

	Categorical variable						
Numeric variable		DMN			DMN + GP		
	Control (*n*)^a^	1st to 2nd wk (*n*)^b^	3rd to 4th wk (*n*)^b^	5th to 6th wk (*n*)^b^	2nd wk (*n*)^c^	3rd to 4th wk (*n*)^c^	5th to 6th wk (*n*)^c^

Albumin (g/dL)	4.6 ± 0.3 (23)	3.9 ± 0.7 (07)	3.5 ± 0.6 (11)	3.2 ± 0.1 (07)	3.7 ± 0.5 (06)	4.0 ± 0.7 (12)	4.3 ± 0.6 (14)
GPT (U/L)	58.8 ± 19.6 (23)	459.5 ± 78.5 (08)	566.6 ± 313.5 (11)	763.6 ± 405.2 (07)	235.0 ± 128.6 (06)	207.0 ± 111.7 (12)	262.5 ± 386.2 (14)
GOT (U/L)	101.9 ± 30.4 (23)	661.5 ± 134.4 (08)	1006.1 ± 749.6 (11)	1572.9 ± 965.3 (07)	271.3 ± 106.0 (06)	190.4 ± 114.5 (12)	218.0 ± 259.6 (14)
Bilirubin (mg/dL)	0.11 ± 0.04 (24)	0.72 ± 0.53 (08)	1.00 ± 0.74 (11)	1.13 ± 1.00 (07)	0.25 ± 0.14 (06)	0.25 ± 0.20 (12)	0.3 ± 0.32 (14)
AKP (KA)	45.9 ± 7.5 (12)	600.8 ± 93.0 (04)	668.3 ± 222.0 (03)	468 ± 12.7 (02)	298.2 ± 65.8 (04)	353.3 ± 62.3 (08)	374.0 ± 88.3 (11)
LDH (IU/L)	280.5 ± 49.0 (11)	414.8 ± 102.7 (04)	562.0 ± 120.8 (03)	853.5 ± 91.2 (02)	352.0 ± 21.9 (04)	378.6 ± 36.1 (08)	329.5 ± 67.6 (11)
Globulin (g/dL)	7.0 ± 0.4 (11)	6.65 ± 0.1 (02)	5.0 ± 0.84 (04)	3.6 ± 03 (02)	5.9 ± 0.6 (04)	5.7 ± 0.5 (08)	5.9 ± 1.0 (11)
Triglyceride (mg/dL)	148.0 ± 33.7 (12)	151.3 ± 107.3 (04)	180.9 ± 144.4 (07)	102.8 ± 35.1 (05)	73.0 ± 26.9 (02)	96.5 ± 29.9 (08)	98.3 ± 32.2 (11)
AFP (ng/dL)	0.26 ± 0.06 (10)	0.40 ± 0.19 (04)	0.38 ± 0.05 (04)	0.35 ± 0.07 (02)	0.20 ± 0.00 (02)	0.28 ± 0.13 (08)	0.26 ± 0.08 (10)
CHOL (mg/dL)	83.2 ± 0.14.1 (12)	76.5 ± 7.9 (04)	69.5 ± 12.8 (06)	66.6 ± 17.5 (05)	60.0 ± 5.0 (04)	65.9 ± 15.5 (08)	70.8 ± 18.6 (11)
BUN (mg/dL)	27.2 ± 6.0 (12)	32.5 ± 3.9 (04)	36.25 ± 2.2 (04)	30.5 ± 4.9 (02)	18.8 ± 3.9 (04)	20.9 ± 2.2 (08)	30.6 ± 23.0 (11)
ACP (mg/dL)	2.4 ± 0.6 (12)	1.9 ± 0.6 (04)	6.2 ± 1.1 (04)	8.2 ± 0.6 (02)	3.8 ± 0.6 (04)	5.0 ± 1.5 (08)	3.7 ± 1.1 (11)
PT (sec)	13.1 ± 1.1 (22)	18.5 ± 4.0 (08)	19.7 ± 4.3 (09)	21.5 ± 4.6 (06)	16.1 ± 2.6 (06)	17.2 ± 5.4 (10)	16.3 ± 2.9 (13)
PLT (10^3^/mL)	871.5 ± 191.8 (24)	406.6 ± 71.7 (07)	300.2 ± 164.7 (11)	228.6 ± 302.4 (07)	535.3 ± 137.9 (06)	548.9 ± 259.1 (12)	704.5 ± 301.1 (14)

^a^: Mean ± SD of value from 1st to 6th week in control groups.

^b^: Mean ± SD of value from 1st to 2nd, 3rd to 4th or 5th to 6th week in DMN treatment groups.

^c^: Mean ± SD of value from 2nd, 3rd to 4th or 5th to 6th week in DMN+GP treatment groups.

*n*: number of rats.

**Table 3:** Therapeutic effects of *Graptopetalum paraguayense* (GP) on DMN-induced liver fibrosis in rats via biochemical analysis (2).

Numeric variable	*p* ^ a^			Categorical variable^b^		
	Drug^c^	Week^d^	Drug × Week	1st to 2nd wk	3rd to 4th wk	5th to 6th wk
Albumin	0.1285	0.3895	0.2342	−0.17	0.38	0.83
GPT	**0.0003**	0.6769	0.7826	−225	−360	−501
GOT	**<0.0001**	0.3298	0.2164	−390	−816	−1355
Bilirubin	**<0.0001**	0.6728	0.7539	−0.48	−0.75	−1
AKP	**<0.0001**	0.6172	0.1147	−303	−230	−107
LDH	**<0.0001**	**0.0132**	**0.0013**	−63	−141	−363
Globulin	0.1341	**0.029**	**0.0254**	−0.75	0.58	1.65
Triglyceride	0.0658	0.4116	0.355	−78	−84	−5
AFP	**0.0031**	0.8992	0.6289	−0.2	−0.1	−0.1
CHOL	0.324	0.9847	0.3248	−16.5	−3.6	4.2
BUN	**0.0444**	0.5896	0.4293	−13.8	−13.6	−1.6
ACP	**0.097**	**0.0004**	**0.0006**	1.8	−0.7	−3.6
PT	**0.0285**	0.7278	0.9627	−2.4	−2.5	−5.3
PLT	**0.0008**	0.3879	0.4526	129	249	476

^a^: Significant correction is marked in bold at the 0.05 level by two-way ANOVA.

^b^: Average_DMN + GP_-Average_DMN_ in 1st to 2nd, 3rd to 4th or 5th to 6th week.

^c^: DMN or DMN + GP treatment groups.

^d^: 1st to 2nd, 3rd to 4th or 5th to 6th week.
