# Supplementary figures and images for: Evaluation of the Chinese Medicinal Herb, Graptopetalum paraguayense, as a Therapeutic Treatment for Liver Damage in Rat Models
Source: Evid Based Complement Alternat Med. 2012 Jul 2;2012:256561. doi: 10.1155/2012/256561 (PMC3395323; doi:10.1155/2012/256561)

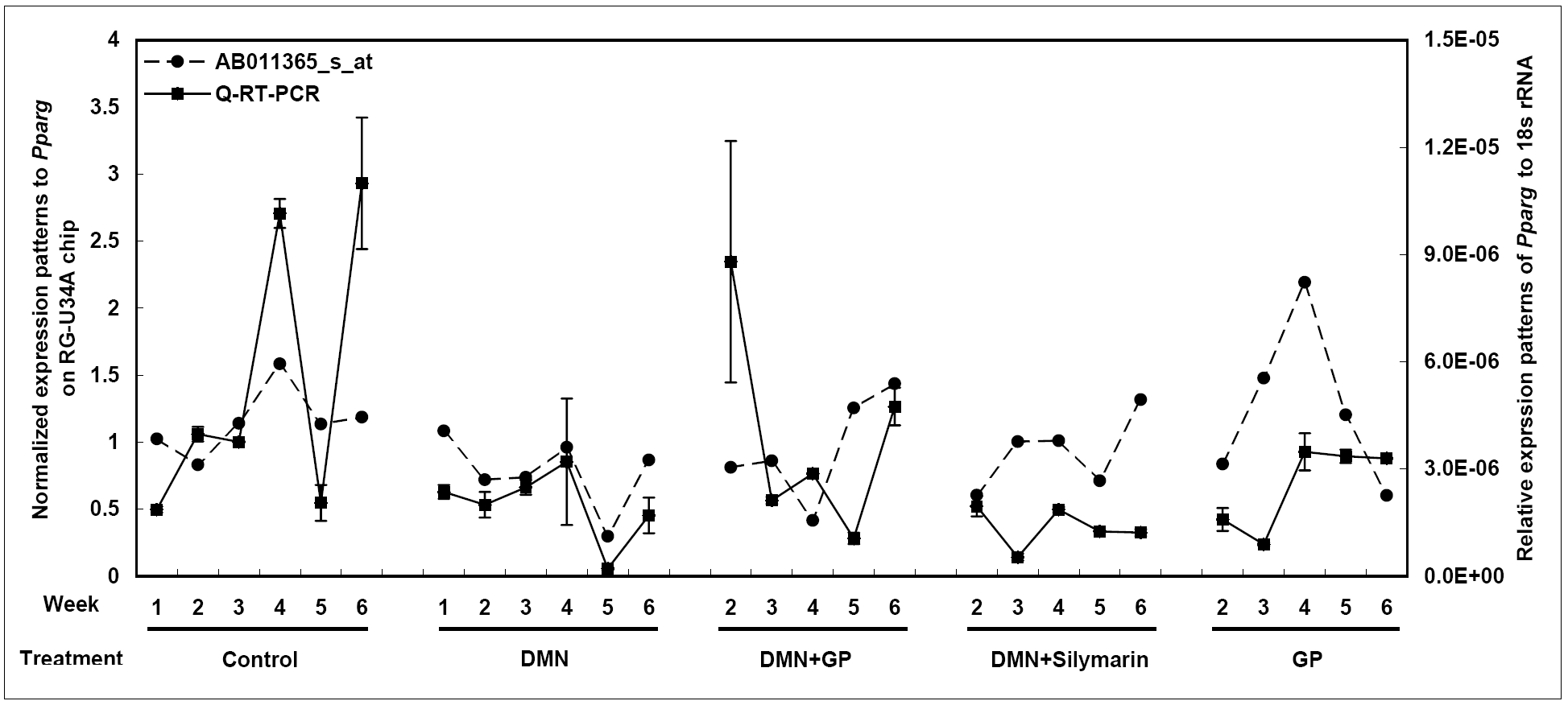


**Supplemental figure 1. Su *et al***

Supplement: Supplementary file 1 — Supplementary Figure 1: Down-regulated expression of peroxisome proliferator-activated receptor, gamma (Pparg) was found in DMN-treated rat liver, consistent with the hypothesis that down regulation of Pparg may be connected to liver inflammation and fibrosis mechanisms. Microarray and Q-RT-PCR results indicated that Pparg expression recovered after GP treatment, suggesting potential for protecting or preventing liver damage. Supplementary Figure 2: Rats in the DMN-damaged group treated with GP fared much better than those treated with Silymarin, especially in the sixth week by hierarchical clustering analysis. Supplementary Table: Statistical analysis indicates that these 168 genes might serve as therapeutic target genes and GP could regulate the gene expression patterns better than Silymarin. [file 256561.f1.doc]
