# Supplementary material for: Evaluation of the Chinese Medicinal Herb, Graptopetalum paraguayense, as a Therapeutic Treatment for Liver Damage in Rat Models
Source: Evid Based Complement Alternat Med. 2012 Jul 2;2012:256561. doi: 10.1155/2012/256561 (PMC3395323; doi:10.1155/2012/256561)
Supplement: Supplementary file 2 [file 256561.f2.doc]

**Supplemental figure 2. Su *et al***
